# Adiponectin serum level is an independent and incremental predictor of all‐cause mortality after transcatheter aortic valve replacement

**DOI:** 10.1002/clc.23892

**Published:** 2022-08-06

**Authors:** Jeroen Walpot, Paul van Herck, Valerie Collas, Liene Bossaerts, Caroline M. Van de Heyning, Tom Vandendriessche, Hein Heidbuchel, Inez Rodrigus, Christophe De Block, Gary R. Small, Johan Bosmans

**Affiliations:** ^1^ Department of Cardiology University Hospital Antwerp Edegem Antwerp Belgium; ^2^ Department of Cardiology ZorgSaam Hospital Terneuzen The Netherlands; ^3^ Faculty of Health Sciences and Medicine University of Antwerp Wilrijk Belgium; ^4^ Faculty of Biomedical Sciences University of Antwerp Wilrijk Belgium; ^5^ Department of Cardiac Surgery University Hospital Antwerp Antwerp Edegem Belgium; ^6^ Department of Endocrinology‐Diabetology and Metabolism Antwerp University Hospital Edegem Antwerp Belgium; ^7^ Division of Cardiology University of Ottawa Heart Institute Ottawa Ontario Canada

**Keywords:** adiponectin, all‐cause mortality, cytokines, frailty, psoas muscle low‐density muscle fraction, STS score, transcatheter aortic valve replacement

## Abstract

**Background:**

Quantifiable biomarkers may be useful for a better risk and frailty assessment of patients referred for transcatheter aortic valve implantation (TAVI).

**Hypothesis:**

To determine if adiponectin serum concentration predicts all‐cause mortality in patients undergoing TAVI.

**Methods:**

77 consecutive patients, undergoing TAVI, were analyzed. The CT axial slices at the level of the fourth lumbar vertebra were used to measure the psoas muscle area, and its low‐density muscle fraction (LDM (%)). To assess the operative risk, the STS (Society of Thoracic Surgeons Predicted Risk of Mortality) score, Log. Euroscore, and Euroscore II were determined. A clinical frailty assessment was performed. ELISA kits were used to measure adiponectin serum levels. We searched for a correlation between serum adiponectin concentration and all‐cause mortality after TAVI.

**Results:**

The mean age was 80.8 ± 7.4 years. All‐cause mortality occurred in 22 patients. The mean follow‐up was 1779 days (range: 1572–1825 days). Compared with patients with the lowest adiponectin level, patients in the third tertile had a hazards ratio of all‐cause mortality after TAVI of 4.155 (95% CI: 1.364–12.655) (*p* = .004). In the multivariable model, including STS score, vascular access of TAVI procedure, LDM (%), and adiponectin serum concentration, serum adiponectin level, and LDM(%) were independent predictors of all‐cause mortality after TAVI (*p* = .178, .303, .042, and .017, respectively). Adiponectin level was a predictor of all‐cause mortality in females and males (*p* = .012 and 0.024, respectively).

**Conclusion:**

Adiponectin serum level is an independent and incremental predictor of all‐cause mortality in patients undergoing TAVI.

**Abbreviations**
AS: aortic stenosis
AVA: aortic valve area
CT: computed tomography
LDM(%): low‐density muscle expressed as a percentage of the PMA
PMA: P[soas muscle area
STS‐score: Society of Thoracic Surgeons Predicted Risk of Mortality score
TAVI: transcatheter aortic valve implantation

## 1 INTRODUCTION

Over the last two decades, transcatheter aortic valve implantation (TAVI) has evolved from an experimental procedure to an appropriate treatment of symptomatic aortic stenosis in patients at high operative risk.^1^, ^2^ Nonetheless, the appropriate selection of patients, who will benefit from TAVI, remains challenging.

It has been demonstrated that conventional clinical cardiac surgery risk assessment, by EuroSCORE II^3^, logistic EuroSCORE,^3^ and STS Score,^4^ predicts mortality after TAVI. Clinical frailty measurements have been demonstrated to be predictive of mortality after TAVI.^5^, ^6^ More recently, imaging studies explored if computed tomography (CT) derived measurements of visceral adipocyte tissue (VAT), subcutaneous adipocyte tissue (SAT), and psoas muscular circumferential surface area (CSA psoas) can be used as surrogate markers of frailty or sarcopenia to predict all‐cause mortality in patients, scheduled for surgery or TAVI.^7^, ^8^

Plasma adiponectin has been proposed as a potential biomarker of frailty.^9^, ^10^, ^11^ Adiponectin is recognized as a plasma cytokine that is released from adipose and skeletal muscle; plasma levels are increased in patients with decreased skeletal muscle mass, weight loss, and reduced physical activity.^11^ Elevated adiponectin levels are also noted in elderly cognitively impaired patients, and adiponectin has been proposed as a biomarker to detect earlier cognitive decline in frail elderly patients.^12^ There may be sex differences between adiponectin levels, frailty and mental status.^13^ In females, there is a negative relationship noted between elevated adiponectin levels and both frailty and anxiety: such that high levels of adiponectin are associated with low body mass index and low levels of anxiety. Whereas in males, the relationship is more complex; increasing adiponectin levels were associated with increased fat mass but decreased lean mass. Overall, though, there does appear to be a negative correlation between lean mass and adiponectin levels in both sexes.^13^ Thus, in elderly patients undergoing TAVR, adiponectin could provide useful prognostic data in terms of potential cognitive decline postprocedure or underlying frailty periprocedure. Adiponectin assessment could also help to determine those patients more likely to benefit from post‐TAVR rehabilitation to improve outcomes.

Therefore, this study sought to determine whether plasma adiponectin levels could predict all‐cause mortality after TAVI. We tested the ability and relative strength of adiponectin levels to predict all‐cause mortality after TAVI in a multivariable model, that included clinical operative risk assessment, frailty assessment, vascular access of the TAVI procedure, CT imaging variables (LMD (%)) and adiponectin serum concentration.

## 2 METHODS

### 2.1 Patient selection

Between January 2014 and June 2016, patients referred for TAVR were included in the analysis. The recruitment of patients to this study partially overlapped with another ongoing frailty study.^14^ Inclusion criteria included having a serum sample of adiponectin, severe aortic stenosis, and undergoing TAVR CT. Exclusion criteria included the inability to obtain informed consent.

The protocol was approved by the local research ethics board. The study has been carried out in accordance with The Code of Ethics of the World Medical Association (Declaration of Helsinki).

#### 2.1.1 Data collection

The UZAntwerp TAVI registry is a comprehensive electronic database of patients undergoing TAVI. Patient baseline characteristics, TAVI procedural information, and outcome data (all‐cause mortality and day of death) were retrieved from the registry (Table 1). In addition, the registry also recorded preoperative assessment risk scores (the logistic EuroSCORE,^3^ EuroSCORE II,^15^ and STS scores^4^) and clinical frailty scores (Fried score,^16^ the FRAIL scale,^17^ the Barthel index,^18^ and the Katz score^19^).

**Table 1:** Patient demographics

Variables	Total study population (*N* = 77)	All‐cause mortality after TAVI – (*n* = 55)	All‐cause mortality after TAVI + (*n* = 22)	*p* value
Age	80.79 ± 7.35	79.82 ± 7.29	83.23 ± 7.08	.066
Gender (male)	34 (44.2)	19 (34.5)	15 (68.2)	.007
Height	165.35 ± 7.94	164.95 ± 6.91	166.23 ± 10.11	.339
Weight	72.84 ± 14.38	73.02 ± 14.42	72.38 ± 14.60	.862
BMI	26.72 ± 4.65	26.83 ± 4.72	26.02 ± 4.63	.691
BSA	1.82 ± 0.20	1.82 ± 0.19	1.82 ± 0.21	.963
Hospitalization(days)* (*n* = 74)	9.58 ± 5.50	8.67 ± 2.85	12.21 ± 9.42	.015
TTE: AS severity assessment (*n* = 62)*				
AVA (cm^2^)	0.69 ± 0.26	0.69 ± 0.28	0.71 ± 0.24	.792
Peak grad (mm Hg)	71.02 ± 25,52	73.73 ± 24.88	64.39 ± 22.95	.176
Mean grad (mmHg)	46.31 ± 17.57	47.79 ± 17.78	42.78 ± 17.04	.314
LVEF**	51.48 ± 15.60	54.75 ± 13.62	46.22 ± 17.98	.093
Vascular access				
Transfemoral access	63 (81.8)	48 (87.3)	15 (68.2)	.050
Comorbidities				
PCI	20 (26.0)	12 (21.8)	8 (36.4)	.189
CABG	27 (35.1)	18 (32.7)	9 (40.9)	.597
MI	8 (10,4)	4 (7.3)	4 (18.2)	.215
CAD	7 (9.1)	5 (9.1)	2 (9.1)	.684
PVD	11(14.3)	7 (12.7)	4 (18.2)	.537
PM	9 (11,7)	6 (10.9)	3 (13.6)	.709
Stoke/TIA	11 (14.3)	8 (14,5)	3 (13.6)	.918
Diabetes mellitus	15 (19.5)	11 (20.0)	4 (18.2)	.856
NYHA class (*n* = 76)*				.185
II	4 (5.3)	4 (7.4)	0 (0.0)	
III	64 (84.2)	46 (85.2)	18 (81.8)	
IV	8 (10.5)	4 (7.4)	4 (18.2)	
Valve surgery	8 (10.4)	8 (14,5)	0 (0.0)	.059
COPD	7 (9.1)	5 (9.1)	2 (9.1)	.684
Risk assessment scores				
Logistic EuroSCORE	18.40 ± 12.51	16.49 ± 11.12	23.19 ± 14.67	.033
EuroSCORE II	7.33 ± 7.95	6.18 ± 5.00	10.18 ± 12.34	.042
STS Score	4.85 ± 3.08	4.19 ± 2.28	6.49 ± 4.11	.003
Frailty assessment				
Fried score + (*n* = 54)*	15 (27.8%)	8 (22.9%)	7 (36.8%)	.273
FRAIL + (*n* = 69)*	16 (23.2%)	13 (27.1%)	3 (14.3%)	.246
Barthel ind. (*n* = 77)*	89.75 ± 9.59	88.95 ± 10.32	91.67 ± 7.74	.257
Katz score + (*n* = 61)*	20 (32.8)	14 (32.6)	6 (33.3)	.953
Laboratory results				
Tot. chol. (mg/dl)	167.82 ± 46.55	117.94 ± 48.21	156.34 ± 40.65	.194
HDL chol. (mg/dl)	53.49 ± 16.54	52.54 ± 14.47	56.11 ± 21.56	.436
LDL chol. (mg/dl)	93.56 ± 37.41	96.50 ± 39.12	85.39 ± 31.78	.240
Albuminemia (g/l)	29.63 ± 4.74	29.76 ± 4.39	29.26 ± 5.69	.731
Uric acid (mg/dl)	5.99 ± 1.88	5.88 ± 1.65	6.33 ± 2.14	.360
eGFR (ml/min)	56.4 ± 19.9	57.2 ± 20.0	54.6 ± 20.1	.602

Abbreviations: AS, aortic stenosis; AVA, aortic valve area; BMI, body mass index; BSA, body surface area; grad, gradient; CABG, coronary artery bypass grafting; CAD, coronary artery disease; COPD, chronic obstructive disease; HDL chol., high‐density lipoprotein; LDL chol., low‐density lipoprotein; LVEF, left ventricular ejection fraction; MI, myocardial infarction; NYHA class, New York Heart Association class; PCI, percutaneous coronary intervention; PM, pacemaker; PVD, peripheral vascular disease; STS score, Society of Thoracic Surgeons score; TAVI, transcatheter aortic valve implantation; TIA, transient ischemic attack; Tot. chol, total cholesterol.

* Incomplete data.

** LVEF: The transthoracic echocardiography was not repeated in our center if it was done by the referring center. In many reports, the description of the LV systolic function was qualitative and could not be entered in the quantitative analyses. In total LV EF% was available in 29 patients.

### 2.2 TAVI scanning protocol

The CT scanning protocol has been previously described.^14^ Combined prospective ECG‐triggered cardiac CT, chest, abdominal, and intrapelvic CT imaging acquisition to select the appropriate bioprosthetic size and assess the vascular access is part of the routine workup before TAVR.

All CT scans were performed on a dedicated CT scanner (GE lightspeed; General Electric Company, Easton Turnpike). Images were acquired using 80–120 kVp and automated tube current modulation.

A triphasic intravenous radiology contrast (Iomeron 350) injection protocol was used: 80 ml contrast agent at 4 ml/s, followed by 60 ml contrast agent at 4 ml/s plus 60 ml 0.9%NaCl solution at 2 ml/s, followed by 28 ml 0.9% NaCl solution at 2 ml/s).^14^

### 2.3 Assessment of the CT measured psoas muscle variables

The acquisition protocol of the psoas muscle variables has been described previously.^14^ In summary, the psoas muscle area (PMA) was measured by contouring the psoas muscle border on a CT axial slice at the fourth lumbar vertebra. A filter, selecting the voxels with attenuation values between 0 HU and 100 Hounsfield Units (HU), determined the area attributed to the psoas muscle. Subsequently, the low‐density muscle portion (LDM(%)) was measured by selecting the voxels ranging from 0 HU to 29 HU within the psoas muscle area. LDM% expresses LDM as a fraction of the PMA, therefore not requiring adjustment to body surface area (BSA) (Figure S1).

### 2.4 Adiponectin serum concentration measurement

The serum concentration of adiponectin was measured using an enzyme‐linked immunosorbent assay (ELISA) from blood samples from the TAVI‐biobank. The Human HMW Adiponectin/Acrp30 Quantikine® ELISA‐kit (R&D Systems) was used to determine the serum adiponectin concentration.

The adiponectin concentration was measured twice, and the results were averaged. The coefficient of variation (CV), which is the ratio of the standard deviation/mean of both measurements, was calculated. The results of the adiponectin serum concentration measurements were rejected if the CV exceeded 15%.

### 2.5 Statistical analysis

Continuous variables are presented as mean ± standard deviation, and categorical variables are presented as frequencies. Continuous variables were compared using Student's *t*‐test and Chi‐square tests were used to compare categorical variables.

Univariable Cox hazard analyses were performed to determine the hazard ratios of the clinical frailty assessment variables, the operative risk stratification variables, vascular access of the TAVI procedure, LDM (%), and adiponectin serum concentration for all‐cause mortality after TAVI.

The aforementioned variables were eligible for inclusion in the multivariable analysis if their *p*‐value ≤ .2 in the univariable analysis. If several operative risk stratification variables or clinical frailty variables were eligible for inclusion in the multivariable analysis, the variable with the lowest *p*‐value was selected to represent perioperative risk and clinical frailty assessment, respectively. Multivariable Cox hazard analyses were performed to determine the hazard ratios of the adiponectin levels for all‐cause mortality after TAVI. Subgroup analyses were performed to test whether adiponectin serum concentration predicts all‐cause mortality after TAVI in both sexes. Risk‐adjusted Kaplan Meier analysis was performed for adiponectin tertile groups.

Receiver operating characteristic (ROC) curves of Adiponectin concentration were performed to calculate the area under the curve.

Pearson correlations were used to test for a correlation between the Barthel frailty score and LDM%. All analyses were performed in SPSS Version 25 (IBM), and statistical significance was defined as *p* < .05.

## 3 RESULTS

77 patients met the inclusion criteria for the study. However, six patients were excluded from the analyses involving LDM(%) due to insufficient CT data.

### 3.1 Patient characteristics

Thirty‐four (44.2%) patients were male (Table 1). The mean age was 80.8 ± 7.3 years. Mean STS score was 4.85 ± 3.08. The FRAIL scale was positive in 23.2%. 81.8% underwent transfemoral TAVI. The mean duration of hospitalization was 8.67 ± 5.06 days and 12.21 ± 9.42 days in patient without and with mortality, respectively (*p* = .015).

### 3.2 Follow‐up duration

Patients were followed for a mean of 1779.0 ± 81.2 days. The median follow‐up was 1825. Twenty‐two deaths occurred during the follow‐up period.

### 3.3 Distribution of patient characteristics among patients with and without mortality post‐TAVI

Statistically significant differences in sex, hospital stay, operative risk assessment score, and psoas muscle assessment were observed between patients with and without mortality after TAVI (Tables 1 and 2, *p* < .05). A trend toward a statistically significant difference between survivors and nonsurvivor was observed for age, LVEF, vascular access route of TAVI‐procedure (*p* = .066, *p* = .093, *p* = .050, and *p* = .059, respectively).

Frailty scores (frail according to Fried score, FRAIL score, Barthel index, and Katz score) failed to demonstrate statically significant differences between survivors and non‐survivors (*p* = .273, .246, .257, and .953, respectively).

### 3.4 Serum adiponectin

Compared with survivors, the serum adiponectin concentration was statistically significantly higher in patients with mortality after TAVI (Table 2).

**Table 2:** Adiponectin serum levels and CT‐derived psoas muscle variables

Variable	Mean value ± SD	Mortality ‐	Mortality +	*p* value
**Adiponectin**				
Adiponectin (ng/ml)	8766.6 ± 6562.6	7323.3 ± 5938.4	12374.8 ± 6788.8	.002
Adiponectin tertiles	*n*	*n*	*n*	.004
1st	24	20	4	
2nd	26	22	4	
3rd	27	13	14	
**Psoas muscle**				
CSA PM (mm²/m²) *n* = 71	814.26 ± 211.42	813,08 ± 225.22	817.27 ± 176.57	.947
CSA LDM % (*n* = 71)	24.84 ± 6.37	23.67 ± 6.73	27.76 ± 4.24	.014

Abbreviations: CSA, circumferential surface area indexed to body surface area (mm^2^/m^2^) or expressed as a percentage of the total muscle CSA (%); LDM, low‐density muscle fraction of the PM; PM, psoas muscle; SD, standard deviation.

The mean serum adiponectin level was 9733.6 ± 7076.3 ng/ml and 7866.6 ± 5847.7 ng/ml in females and males, respectively (*p* = .215).

### 3.5 Correlations between adiponectin serum level and LDM(%)

No correlation between adiponectin serum level and LDM(%) was found (Pearson correlation: .077; *p* = .526). A subgroup analysis in males and females failed to show a correlation between adiponectin serum level and LDM(%) (Pearson correlation: .074 and −.174; *p* = .526 and .295, respectively).

### 3.6 Survival analysis

Univariable predictors of survival were calculated to determine those to be used in a multivariable cox regression analysis (Table 3). The clinical frailty assessment variables (Fried score, Frail score, Barthel index, and Katz score) were not suitable for inclusion in the model (*p*‐value >.2 in univariable analysis). The multivariable Cox regression survival model, including STS score, vascular access of TAVI procedure, LDM(%) tertiles, and adiponectin tertiles, demonstrated that adiponectin concentration (*p* = .017) and LDM(%) (*p* = .042) are independent predictors of reduced survival after TAVI. Compared to patients in the conjugated first and second adiponectin serum level tertile, patients in the third tertile had a hazard ratio (HR) of 4.6995 (95%CI: 1.055–20.924) (*p* = .004) (Table 3B).

**Table 3:** Univariable (Table 3A) and multivariable (Table 3B) Cox hazard regressions for all‐cause mortality after TAVI

Table 3A: Univariable Cox hazard regressions for all‐cause mortality after TAVI				
Variable	Hazard ratio	95% CI lower bound	95% CI upper bound	*p*‐value
A. **Frailty assessment**				
Fried score	1.808	0.711	4.596	.215
FRAIL scale	0.536	0.158	1.821	.310
Barthel index	1.024	0.969	1.081	.401
Katz core^a^	1.026	0.385	2.734	.959
B. **Preoperative assessment score systems**				
Log. Euroscore	1.041	1.010	1.073	.009
Euroscore II	1.067	1.025	1.111	.002
STScore	1.173	1.060	1.298	.002
C. **Vascular access** *				
Transfemoral versus non‐transfemoral	2.739	1.115	6.731	.028
D. **Cytokines**				
Adoponectin				.004
1st tertile	1	0.230	3.675	.919
2nd tertile	0.919	1.364	12.655	.012
3rd tertile	4.155			
Adiponectin				
1st−2nd tertile	1	1.814	10.373	.001
3rd tertile	4.338			
E. **Psoas muscle attenuation**				
CSA Poas muscle (mm^2^/m^2^)	1.000	0.998	1.002	.866
LDM (%)				.065
First tertile	1	1.301	27.989	.022
Second tertile	6.035	1.153	24.760	.032
Third tertile	5.344			
LDM (%)				
1st tertile	1	1.313	24.463	.020
2nd−3rd tertile	5.668			
F. **Biochemical/metabolic laboratory results at baseline**				
eGRF (ml/min)	0.993	0.973	1.014	.532
Total cholesterol	0.992	0.980	1.004	.156
HDL cholesterol	1.010	0.981	1.040	.505
LDL cholesterol	0.991	0.977	1.006	.218
Albumin	0.980	0.887	1.082	.980
Uric acid	1.166	0.907	1.498	.232
G. **Patients characteristics**				
Age	1.062	0.985	1.145	.117
Gender	5.460	1.795	16.612	.003
BMI	0.979	0.875	1.097	.719
BSA	2.959	0.199	43.905	.431
Hospitalization duration	1.057	1.014	1.102	.010
H. **Comorbidities**				
PCI	1.793	0.695	4.628	.227
CABG	1.488	0.587	3.770	.402
MI	2.531	0.831	7.706	.102
CAD	1.128	0.259	4.906	.873
PVD	1.880	0.618	5.717	.266
PM	0.474	0.063	3.565	.469
Stroke/TIA	1.111	0.321	3.839	.868
Diabetes mellitus	0.690	0.200	2.385	.558
Valvular surgery	0.040	0.000	14.339	.283
COPD	0.597	0.079	4.486	.616

**Table 3B: Multivariable Cox hazard regressions for all‐cause mortality after TAVI**				
**Variable**	**Hazard ratio (HR)**	**95%CI Lower bound**	**95%CI Upper bound**	***p*‐value**
STS score	1.117	0.951	1.312	.178
Vasc. Access				
Transfemoral	1	0.575	5.915	.303
Nontransfem.	1.844			
Adiponectin				
1sth−2nd tertile	1	1.226	7.817	.017
3rd tertile	3.096			
LDM (%)				
1st tertile	1	1.055	20.926	.042
2nd−3rd tertile	4.699			

Abbreviations: BMI, body mass index; BSA, body surface area; CAD, coronary artery disease; CABG, coronary artery bypass grafting; CSA, circumferential surface area indexed to body surface area (mm^2^/m^2^) or expressed as a percentage of the total muscle CSA (%); eGFR, glomerular filtration rate (ml/min/m^2^); HDL cholesterol, high density lipoprotein cholesterol; LDL cholesterol, low density lipoprotein; LDM, low density muscle; MI, myocardial infarction; PCI, percutaneous coronary intervention; PM, pacemaker; PVD, peripheral vascular disease; STS score, Society of Thoracic Surgeons score; TAVI, transcatheter aortic valve implantation; TIA, transient ischemic attack.

^a^ Katz frailty score was used a binary system (Katz ≤ 5 or >5).

* Vascular access of the TAVI procedure: transfemoral and non‐transfemoral access are compared. Transfemoral access is the reference category.

**Figure 1:**
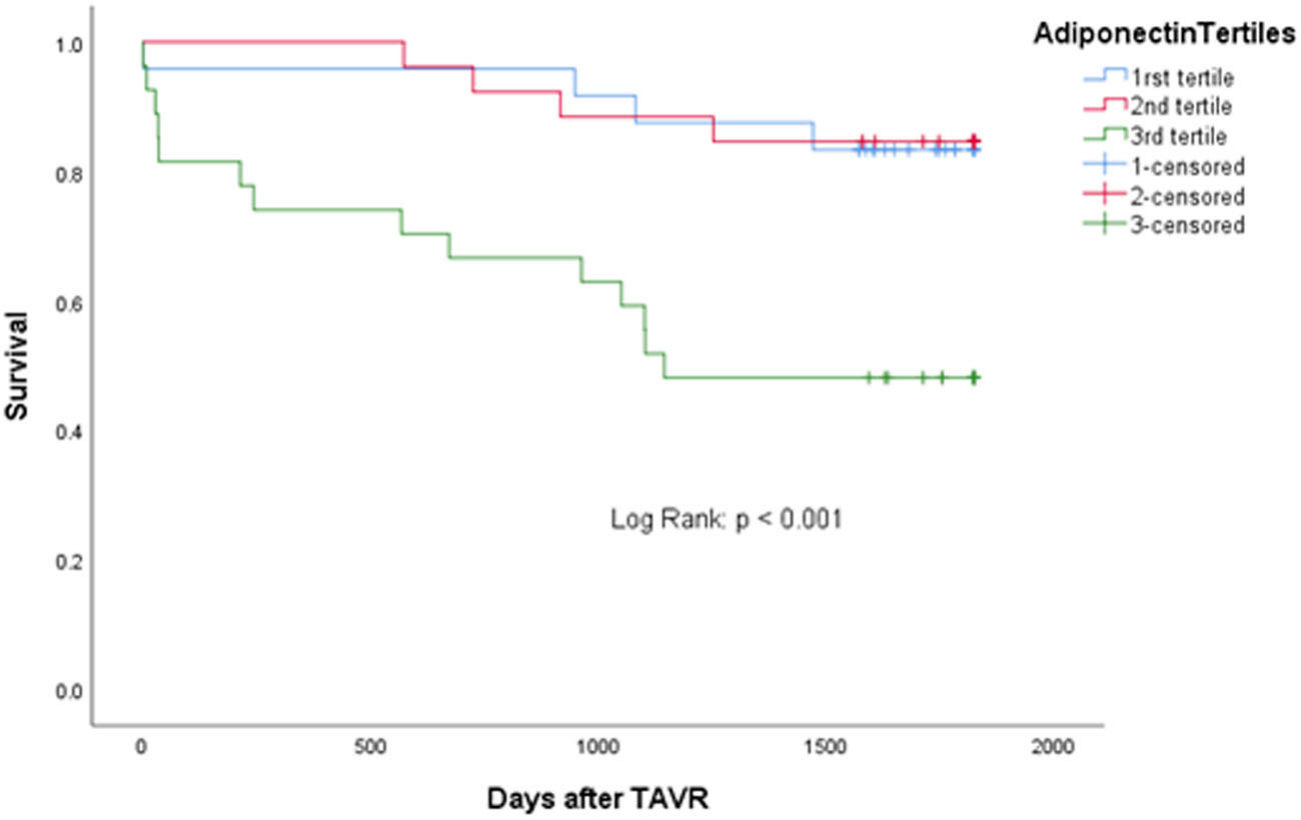
Shows the Kaplan–Meier analysis for adiponectin serum concentration tertile groups (Log Rank *p* < .001). The blue, red and green curve represent the 1st, 2nd and 3rd adiponectin concentration tertile.

Risk adjusted Kaplan–Meier analysis was performed for adiponectin serum concentration tertile groups (Log Rank *p* < .001) (Figure 1).

**Figure 2:**
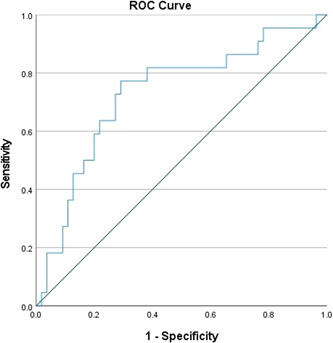
Receiver operating characteristic (ROC) curves of adiponectin concentration with area under the curve (AUC) of 0.730 (95% CI: 0.60–0.86).

Receiver operating characteristic (ROC) curves of adiponectin were performed, with an area under the curve (AUC) of 0.730 (95%CI: 0.60–0.86) (Figure 2).

#### 3.6.1 Subgroup survival analysis in females and males

In both sexes, adiponectin level was associated with survival. Compared to males and females within the conjugated 1st and 2nd adiponectin concentration tertile, males and females within the third tertile had a hazard ratio of death of 3.761 (95%CI: 1.346–10.509) (*p* = .012) and 11.549(95%CI: 1.388–96.062) (*p* = .024), respectively. Figure S2 shows the Kaplan–Meier survival curves for adiponectin serum levels in males and females (Log Rank *p* = .007 and 0.004, respectively).

#### 3.6.2 Supplemental post hoc multivariable analyses

A post hoc multivariable model was tested to search if the addition of metabolic variables may have altered the ability of adiponectin serum levels to predict mortality after TAVI. The introduction of diabetes mellitus, body mass index (BMI), and LDL cholesterol to the multivariable model did not change the overall conclusion that adiponectin is an independent predictor of all‐cause mortality after TAVI (Table S1).

A post‐hoc binary logistic analysis, using the 30‐days mortality after TAVI as the marker for TAVI‐procedure‐related mortality, failed to demonstrate an association between adiponectin level and 30‐days mortality after TAVI (*p* = .997).

## 4 DISCUSSION

Serum adiponectin levels have been proposed as a biomarker for frailty.^9^, ^10^ Due to this feature, serum adiponectin concentration may be a potential predictor of adverse outcome after TAVI. The medical literature on adiponectin in the setting of aortic stenosis is limited. A Medline search entering the terms “adiponectin and aortic stenosis” showed only 23 hits. Most studies searched for associations between adiponectin serum level and mechanical and mechanistic aspects of AS, such as valve calcification, progression of native valve stenosis, degeneration of bioprosthetic valves, and LV remodeling.^20^, ^21^, ^22^, ^23^, ^24^ Little is known about the ability of adiponectin to predict adverse outcome after TAVI due to severe aortic stenosis (AS).

Our study, with a mean follow‐up of 1779 days, demonstrates that adiponectin serum concentration is an independent and incremental predictor of all‐cause mortality after TAVI in males and females.

On the other hand, adiponectin serum level was not a predictor for mortality 30 days after TAVI. This finding may suggest that adiponectin is no predictor of TAVI‐procedure related complications, despite the fact that adiponectin has been associated with aortic valve calcification.

Adiponectin has been associated with protective cardiovascular effects due to its anti‐atherosclerotic and anti‐inflammatory properties and ability to reduce insulin intolerance.^25^ However, there is little data about the role of adiponectin in patients with severe AS.^25^

Prior investigators have considered the potential for adiponectin to prevent adverse events through proposed anti‐inflammatory actions in aortic stenosis.^20^, ^21^, ^22^, ^23^ The role of adiponectin to predict frailty per se was not considered. These investigators selected patients who were younger than our cohort and often were surgical AVR candidates, so patient frailty was less of a concern.^20^, ^21^, ^22^

Our study differs from the aforementioned studies by the chosen endpoint and the variables, included in the multivariable model. Our study sought to answer whether serum adiponectin levels before TAVI predict all‐cause mortality after TAVI. Furthermore, our multivariable model focused on including clinical variables, such as operative risk assessment (STSS), vascular access of the TAVI procedure, and a CT‐derived psoas muscle attenuation variable (as a surrogate marker for sarcopenia).

Nevertheless, it should be noted that our study results do not align well with the current insight that adiponectin has mainly cardioprotective effects, as patients within the highest serum adiponectin concentration had the highest all‐cause mortality.^25^, ^26^ This may reflect competing effects of adiponectin in the elderly, where perhaps its proposed anti‐inflammatory effects are less protective.

On the other hand, our study results are consistent with the findings from frailty studies, demonstrating that adiponectin levels are elevated in elderly cognitively impaired patients.^9^, ^10^, ^11^

In contrast to earlier frailty studies, showing sex differences between adiponectin levels, frailty, and mental status, our results confirmed a strong and independent association between adiponectin serum concentration before TAVI and all‐cause mortality after TAVI in both sexes. Males and females in the third adiponectin serum level had a hazard ratio of death of 3.761 (*p* = .012) and 11.549 (*p* = .024) respectively, compared to their counterparts in the combined first and second tertile.

The finding that adiponectin serum concentration, irrespective of sex, is an independent and incremental predictor of all‐cause mortality after TAVI should encourage further research to determine its role in selecting the patients that will most benefit from a TAVI procedure or intensive rehabilitation programs after TAVI.

CT measured psoas muscle area (PMA) has been proposed as a surrogate marker for sarcopenia. Several studies have demonstrated that PMA is a predictor of mortality after TAVI.^7^, ^8^, ^27^ Authors emphasized the relevance of sex‐adjustment of PMA. A study found that the lowest tertile PMA of patients undergoing TAVI consisted of women, requiring sex adjusted PMA tertiles to search for correlations between PMA and survival after TAVI.^8^ Previously, we demonstrated a statistically significant difference in body surface area (BSA) adjusted LDM between males and females.^14^ When the LDM area was expressed as a fraction (LDM%) of the PMA, no statistically significant sex differences were found. Overall, LDM% outperformed BSA‐adjusted LDM in predicting all‐cause mortality after TAVI.^14^

Given that adiponectin plasma concentration and LDM% were proposed to represent frailty and sarcopenia, we tested for a correlation between both variable but did not demonstrate one. This may suggest that adiponectin better represents other aspects of frailty than sarcopenia.

Selecting the variables for our multivariable model was challenging. Our model builds further on research by others. Historically, studies confirming an association between clinical operative risk assessment, using the EuroSCORE II, the Logistic EuroSCORE, or the STS score were performed first.^28^, ^29^ Subsequently, imaging studies tested the utility of CT measured psoas muscle variables and body adipose tissue distribution to predict outcomes after TAVI, often in multivariable analysis with clinical operative risk assessment.^8^, ^14^ We add adiponectin serum level as a third variable to the model.

Our study results demonstrated that hospitalization duration is a predictor for all‐cause mortality after TAVI. We deliberately did not include hospital stay in our multivariable model as this variable does not contribute to the clinical decision‐making before TAVI implantation.

The vascular access route of TAVI was an independent predictor of all‐cause mortality in the univariable analysis but failed in the multivariable analysis. Given the anti‐inflammatory and anti‐atherosclerotic properties attributed to adiponectin, a correlation between adiponectin level and arterial vascular obstruction could be hypothesized. However, our study sample is too small to elaborate on this hypothesis further. Nevertheless, our study could not demonstrate an association between the adiponectin serum concentration and the TAVI‐procedure‐related 30‐days mortality.

Age, sex, and cachexia have been associated with mortality after TAVI.^30^, ^31^, ^32^ Age and sex are incorporated in the operative risk assessment score systems and thus embedded in our multivariable model.

### 4.1 Study limitations

Three study limitations need to be discussed. First, it could be argued that the small sample size of our study may have altered the study results. On the other hand, the ability of adiponectin levels to predict all‐cause mortality after TAVI in this limited study population indirectly proves the prognostic strength of serum adiponectin levels. Our sample size is small with 77 patients being included in the analysis. The finding of an increased risk for those in the highest tertile of adiponectin is hypothesis‐generating, and further large‐scale prospective studies are required to validate our observations.

All‐cause mortality was used as the outcome variable in our study. This reflected the fact that frailty may lead to death through multiple causes, for example: fracture hip, aspiration pneumonia, and head injury. In this small sample size, further analysis of the potential effects of adiponectin on other causes of mortality such as cardiovascular events or sepsis was not possible.

Adiponectin levels may have been influenced by multiple metabolic factors, such as lipid profile, diabetes, smoking status, and medication use. These potential confounders could only partially be extracted from the database. To mitigate this limitation, diabetes, body mass index (BMI), and LDL cholesterol were introduced in a supplemental multivariable model (Supplemental Table 1). The overall conclusion that adiponectin is an independent predictor of all‐cause mortality after TAVI remained unchanged. It should be emphasized that strong predictors of mortality after TAVI, such as age and sex are part of the STS Score and thus included in the multivariable model.

## 5 CONCLUSION

Our study, with a mean follow‐up of 1779 days, demonstrates that adiponectin serum concentration is an independent and incremental predictor of all‐cause mortality after TAVI. Further prospective studies are warranted to determine the role of serum adiponectin levels to predict frailty and outcomes in patients with severe AS, who are being considered for TAVI.

## CONFLICTS OF INTEREST

HH: Dr. Heidbuchel did not receive any personal honoraria. He received unconditional research grants through the University of Antwerp and/or the University of Hasselt from Bayer, Boehringer‐Ingelheim, Bracco Imaging Europe, Abbott, Medtronic, Biotronik, Daicchi‐Sankyo, Pfizer‐BMS, and Boston‐Scientific, all outside the scope of this study. JB: Dr. Bosmans is a proctor for Medtronic CoreValve.

## Supporting information


*Supplemental Figure 1* demonstrates the calculation of the portion of Low‐density muscle (LDM%). The slice portraying the 4^th^ lumbar vertebra is selected. Panel A shows the manually contoured psoas area. Subsequently, the filter selects the voxels with Hounsfield Units (HU), ranging between 0 and 29 HU, attributed to LDM (Panel B).

*Supplemental Figure 2* shows the Kaplan Meier survival curves for adiponectin serum levels in males and females (Log Rank p = 0.007 and 0.004, respectively). Compared to males and females within the conjugated 1^st^ and 2^nd^ adiponectin concentration tertile (green curves), males and females within the third tertile (green curves) had a hazard ratio of death of 3.761 (95%CI: 1.346 – 10.509) (p = 0.012) and 11.549(95%CI: 1.388 −96.062) (p = 0.024), respectively.

Supplementary information.

## Data Availability

The data that support the findings of this study are available from Dr. J. Bosmans upon reasonable request.
