# Supplementary figures and images for: Adiponectin serum level is an independent and incremental predictor of all‐cause mortality after transcatheter aortic valve replacement
Source: Clin Cardiol. 2022 Aug 6;45(10):1060–9. doi: 10.1002/clc.23892 (PMC9574742; doi:10.1002/clc.23892)

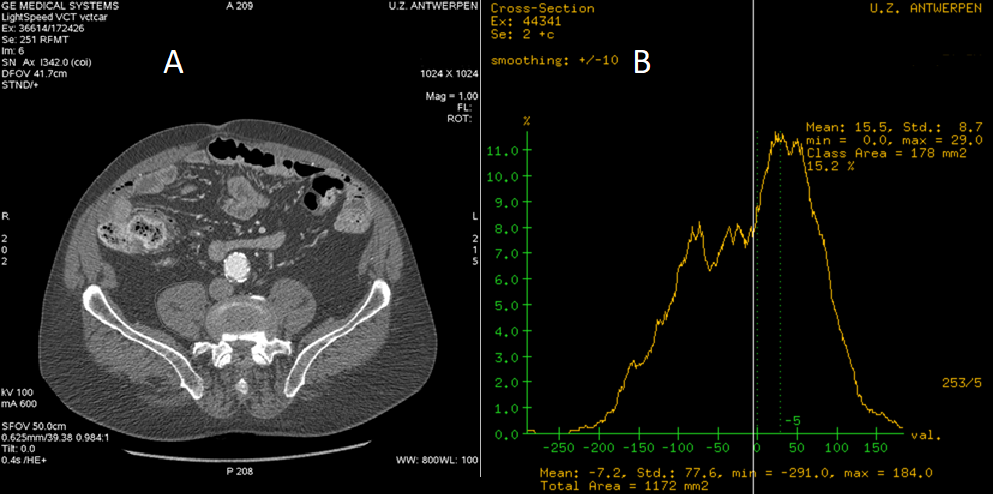

Supplement: Supplementary file 1 — Supplemental Figure 1 demonstrates the calculation of the portion of Low‐density muscle (LDM%). The slice portraying the 4th lumbar vertebra is selected. Panel A shows the manually contoured psoas area. Subsequently, the filter selects the voxels with Hounsfield Units (HU), ranging between 0 and 29 HU, attributed to LDM (Panel B). [file CLC-45-1060-s003.jpeg]

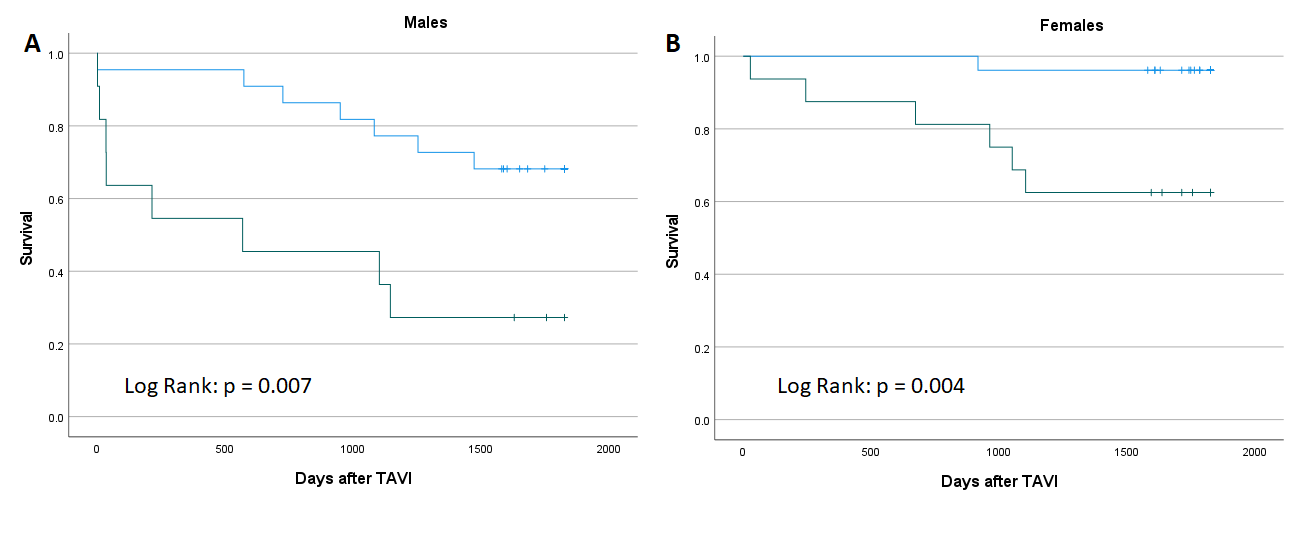

Supplement: Supplementary file 2 — Supplemental Figure 2 shows the Kaplan Meier survival curves for adiponectin serum levels in males and females (Log Rank p = 0.007 and 0.004, respectively). Compared to males and females within the conjugated 1st and 2nd adiponectin concentration tertile (green curves), males and females within the third tertile (green curves) had a hazard ratio of death of 3.761 (95%CI: 1.346 – 10.509) (p = 0.012) and 11.549(95%CI: 1.388 −96.062) (p = 0.024), respectively. [file CLC-45-1060-s002.jpeg]
