# Supplementary material for: Adiponectin serum level is an independent and incremental predictor of all‐cause mortality after transcatheter aortic valve replacement
Source: Clin Cardiol. 2022 Aug 6;45(10):1060–9. doi: 10.1002/clc.23892 (PMC9574742; doi:10.1002/clc.23892)
Supplement: Supplementary file 3 — Supplementary information. [file CLC-45-1060-s001.docx]

| **Table 1 Supplemental: Multivariable Cox hazard analyses: all-cause mortality after TAVR** | | | | |
| --- | --- | --- | --- | --- |
| **Variable** | **Hazard ratio (HR)** | **95%CI Lower bound** | **95%CI Upper bound** | **P value** |
| STSS | 1.133 | 0.947 | 1.354 | 0.172 |
| Vasc. Acces | 4.803 | 1.283 | 17.982 | 0.020 |
| Adiponectin  1st-2nd tertiles  3^rd^ tertile | 1  4.382 | 1.317 | 14.575 | 0.016 |
| LDM (%)  1^sth^ tertile  2^nd^-3rd tertiles | 1  3.676 | 0.760 | 17.793 | 0.106 |
| DM/glucose intolerance | 0.314 | 0.080 | 1.236 | 0.98 |
| LDL cholesterol | 0.993 | 0.977 | 1.011 | 0.455 |
| BMI | 1.064 | 0.920 | 1.231 | 0.403 |

TAVI: transcatheter aortic valve implantation; STS score: Society of Thoracic Surgeons score; Vasc. Access: vascular access of the TAVI procedure; LDM (%): Low density muscle expressed as portion of the total psoas muscle. DM: diabetes mellitus; LDL cholesterol: Low-density lipoprotein cholesterol; BMI: Body mass index; 95%CI: 95% confidence interval.
